# The Hot Quadrate Sign on a Ventilation-Perfusion (V/Q) Scan

**DOI:** 10.7759/cureus.64122

**Published:** 2024-07-09

**Authors:** Kabeer Masih, Pokhraj P Suthar, Sumeet Virmani

**Affiliations:** 1 Department of Diagnostic Radiology and Nuclear Medicine, Rush University Medical Center, Chicago, USA

**Keywords:** chronic dialysis, ct angiogram, v/q scan, quadrate lobe, hot quadrate sign

## Abstract

The hot quadrate sign is defined as an intense arterial enhancement in the hepatic quadrate lobe, most frequently encountered on CT angiograms in patients with central venous occlusion. It has also been described as focal uptake on technetium-99m (Tc99m) sulfur colloid scans. We present an unusual case of focal uptake in the hepatic quadrate lobe on a ventilation-perfusion (V/Q) scan, corresponding to the hot quadrate sign in a 42-year-old patient with chronic kidney disease and central venous occlusion.

## Introduction

The hot quadrate lobe sign, characterized by intense arterial enhancement in the hepatic quadrate lobe, is a critical diagnostic marker predominantly associated with superior vena cava (SVC) or central venous obstruction [1,2]. This condition is typically seen above the level of the azygos arch, where blood flow is rerouted through collateral pathways, including the internal mammary veins, superficial epigastric veins, recanalized paraumbilical vein, and a patent remnant of the umbilical vein [2]. These pathways eventually drain into the left portal vein, resulting in enhanced quadrate lobe visibility. In contrast, obstructions below the azygos arch redirect blood flow into the inferior vena cava via the azygos and hemiazygos veins, typically without causing liver hot spots [2]. Recognition of the hot quadrate lobe sign is essential due to its potential implications in identifying and understanding central venous obstructions. The hot quadrate lobe sign has been observed in the causes of SVC syndrome (such as thoracic neoplasms like lung carcinoma and lymphoma, Vasculo-Behcet's disease, fibrosing mediastinitis, and luetic aneurysm), Budd-Chiari syndrome, and liver masses (including hemangioma, abscess, hepatocellular carcinoma, and focal nodular hyperplasia (FNH)) [3]. This sign has been observed using various imaging modalities, including technetium-99m (Tc99m) sulfur colloid scans, radionuclide venograms, renal scintigraphy, and fluorodeoxyglucose positron emission tomography-computed tomography (FDG PET/CT) [4-7]. Its detection can prevent misinterpretation of the quadrate lobe as a hypervascular lesion, which could lead to unnecessary invasive procedures or misdiagnosis. Understanding the underlying mechanisms and pathways involved in the hot quadrate lobe sign aids in the accurate diagnosis and management of patients with central venous obstructions. It underscores the importance of correlating clinical findings with various imaging modalities to avoid misdiagnosis and ensure appropriate treatment.

## Case presentation

**Figure 1:**
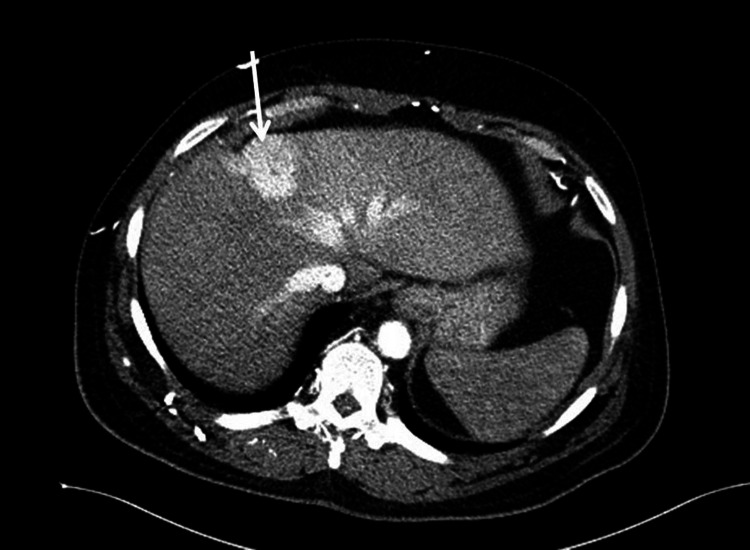
Contrast-enhanced CT of the abdomen demonstrating intense arterial hyperenhancement of the hepatic quadrate lobe

**Figure 2:**
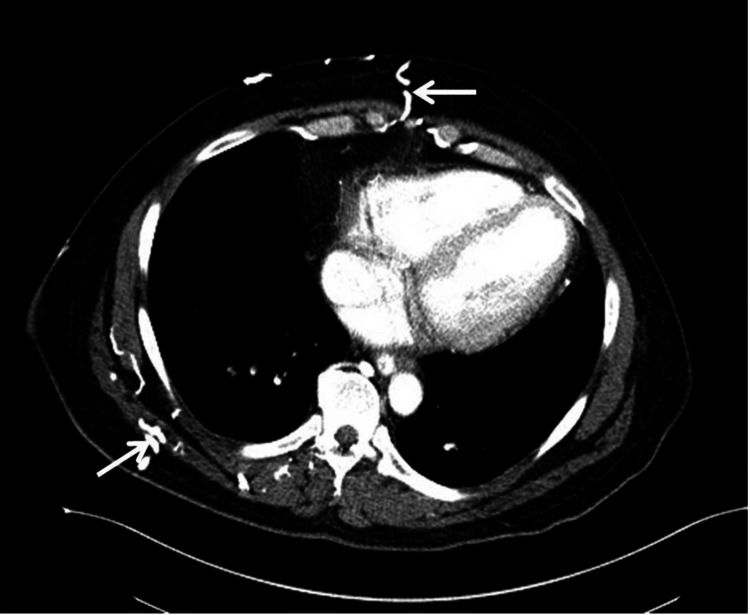
Arterial phase contrast-enhanced CT demonstrating collateral internal mammary vessels along the anterior thoracoabdominal wall, which perfuse the quadrate lobe

**Figure 3:**
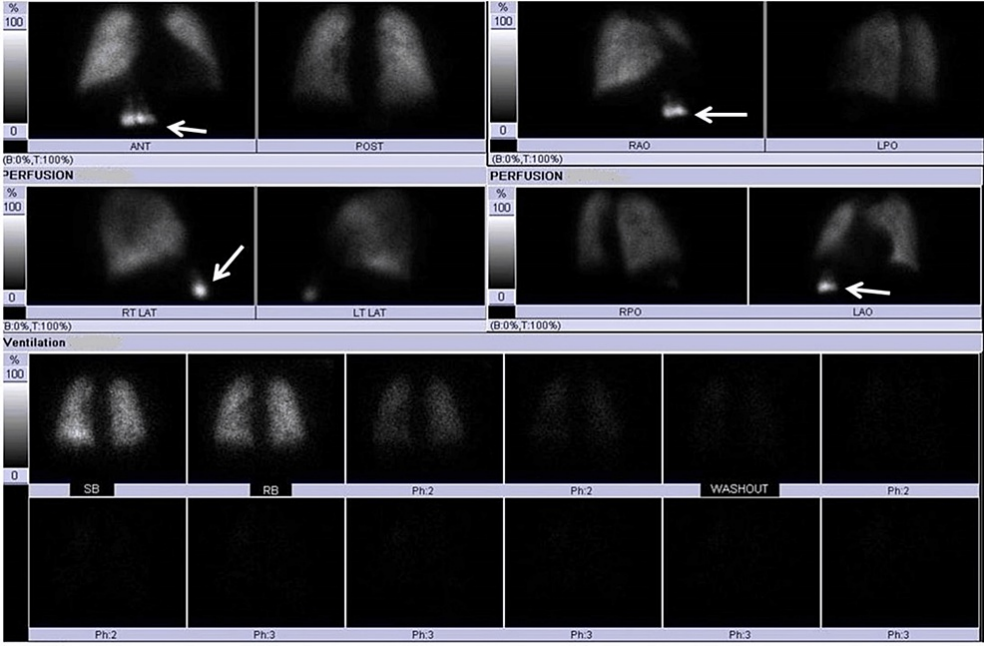
V/Q scan demonstrating focal uptake of Tc99-MAA in the anterior right upper abdomen corresponding to radiotracer uptake by the hepatic quadrate lobe V/Q scan: ventilation-perfusion scan; Tc99m: technetium-99m; MAA: macroaggregated albumin

A 42-year-old male with a past medical history of chronic kidney disease, congestive cardiac failure, deep venous thrombosis, and hyperlipidemia presented to the emergency department with acute left-sided chest pain and dyspnea. The physical exam was unremarkable. Electrocardiogram and troponins were negative for myocardial ischemia. Chest X-ray revealed clear lungs with mild pulmonary vascular congestion. The CT angiogram showed areas of increased arterial enhancement in the quadrate lobe (Figure 1) with extensive collaterals along the anterolateral chest/abdominal wall in continuity with the internal mammary vasculature (Figure 2) from SVC obstruction. A ventilation-perfusion (V/Q) scan was performed and determined to be a “very low” probability for pulmonary embolism. Ventilation imaging showed no abnormal uptake in the abdomen, while the perfusion image showed two adjacent areas of intense extrapulmonary Tc99m-macroaggregated albumin (MAA) activity in the right anterior mid-abdomen (Figure 3), correlating to areas of increased arterial enhancement in the quadrate lobe on a prior CT angiogram. The patient’s clinical history revealed central venous obstruction from central venous catheterization due to the patient’s known end-stage kidney disease. The constellation of these findings is consistent with the "hot quadrate sign," which is seen in patients with SVC obstruction leading to collateralization and portosystemic shunting. The subsequent patient underwent endovascular intervention to relieve central venous obstruction through percutaneous angioplasty and stent placement.

## Discussion

The CT quadrate lobe hot spot sign was initially described by Ishikawa in 1983 [3]. There are various major collateral pathways in the presence of central venous obstruction, including the azygos-hemiazygos system, internal mammary veins, lateral thoracic and superficial thoracoabdominal veins, and vertebral venous plexus. The hot quadrate lobe sign is predominantly seen with SVC/central venous obstruction above the level of the azygos arch, in which case the blood flow will be diverted to internal mammary veins, superficial epigastric veins, recanalized paraumbilical vein, and to a patent remnant of the umbilical vein, which will eventually drain into the left portal vein and lead to quadrate lobe enhancement. An obstruction below the level of the azygos arch causes retrograde venous flow into the inferior vena cava via the azygos and hemiazygos veins without any hot spots in the liver [1,2,8]. The hot quadrate lobe sign has been observed in the causes of SVC syndrome (such as thoracic neoplasms like lung carcinoma and lymphoma, Vasculo-Behcet's disease, fibrosing mediastinitis, and luetic aneurysm), Budd-Chiari syndrome, and liver masses (including hemangioma, abscess, hepatocellular carcinoma, and FNH) [3]. Hot quadrate lobe sign has also been previously demonstrated on Tc99m sulfur colloid scans, radionuclide venograms, renal scintigraphy, and FDG PET/CT [4-7]. The presence of Tc99m-MAA quadrate lobe uptake in our patient is likely due to undiluted concentrations of the peripherally injected radiotracer. The adjunctive use of single-photon emission computed tomography-computed tomography (SPECT/CT) proves beneficial for precise anatomical localization of the area of uptake. On CT angiograms, it manifests as an area of intense, wedge-shaped arterial and venous phase enhancement in the quadrate lobe, which is functionally part of the left hepatic lobe as designated segment IVb in the Bismuth-Couinaud classification system. It became isodence in the delayed phase [8]. Recognition of this entity is crucial in avoiding misinterpretation of this finding as a hypervascular lesion.

## Conclusions

The hot quadrate sign refers to intense arterial enhancement of the hepatic quadrate lobe on CT angiography and is most commonly due to SVC occlusion resulting in shunting of blood flow to the liver via collateral vessels. However, it is important to recognize that the hot quadrate sign can be seen with multiple other imaging modalities, such as V/Q scan as demonstrated in this case. Recognition of this entity is critical in avoiding mischaracterization of the quadrate lobe as a focal hyper-enhancing lesion.
